# Quantification of ETS exposure in hospitality workers who have never smoked

**DOI:** 10.1186/1476-069X-9-49

**Published:** 2010-08-12

**Authors:** Stefanie Kolb, Ulrike Brückner, Dennis Nowak, Katja Radon

**Affiliations:** 1 Institute for Occupational, Social and Environmental Medicine, University Hospital of the Ludwig-Maximilians University Munich, Ziemssenstr. 1, 80336 Munich, Germany

## Abstract

**Background:**

Environmental Tobacco Smoke (ETS) was classified as human carcinogen (K1) by the German Research Council in 1998. According to epidemiological studies, the relative risk especially for lung cancer might be twice as high in persons who have never smoked but who are in the highest exposure category, for example hospitality workers. In order to implement these results in the German regulations on occupational illnesses, a valid method is needed to retrospectively assess the cumulative ETS exposure in the hospitality environment.

**Methods:**

A literature-based review was carried out to locate a method that can be used for the German hospitality sector. Studies assessing ETS exposure using biological markers (for example urinary cotinine, DNA adducts) or questionnaires were excluded. Biological markers are not considered relevant as they assess exposure only over the last hours, weeks or months. Self-reported exposure based on questionnaires also does not seem adequate for medico-legal purposes. Therefore, retrospective exposure assessment should be based on mathematical models to approximate past exposure.

**Results:**

For this purpose a validated model developed by Repace and Lowrey was considered appropriate. It offers the possibility of retrospectively assessing exposure with existing parameters (such as environmental dimensions, average number of smokers, ventilation characteristics and duration of exposure). The relative risk of lung cancer can then be estimated based on the individual cumulative exposure of the worker.

**Conclusion:**

In conclusion, having adapted it to the German hospitality sector, an existing mathematical model appears to be capable of approximating the cumulative exposure. However, the level of uncertainty of these approximations has to be taken into account, especially for diseases with a long latency period such as lung cancer.

## Background

Prior to the implementation of the workplace smoking ban, Environmental Tobacco Smoke (ETS) was, except for exposure to allergens, probably the most important inhalative hazardous substance in terms of number of exposed people in many countries.

Occupational exposure to Environmental Tobacco Smoke (ETS) and subsequent health effects in workers have been frequently investigated in recent studies, especially in Europe and the USA [1-3]. In these, ETS is defined as side-stream smoke which is released by burning cigarettes. Acute symptoms like irritation of eyes and mucous membranes aside, chronic symptoms like exacerbation of chronic bronchial asthma, aggravation of chronic obstructive lung diseases or cardiovascular diseases [4-6] are well known effects on health. Furthermore, several international meta-analyses [7-11] indicate that ETS exposure results in significantly higher relative risks (RR) for lung carcinoma in persons who have never smoked. These studies have found that lung cancer risk is even doubled in these persons, who represent the most exposed subgroup.

Whereas for the majority of the population private ETS exposure in bars or restaurants was negligibly low, hospitality workers were submitted to unlimited exposure to ETS in Germany until 2007. Therefore, ETS exposure may be extremely high in restaurants or bars and in addition, these workplaces are the least protected by law [12,13]. Based upon the results of epidemiological studies, the relation between workplace exposure to ETS and the development of lung cancer in persons who have never smoked seems to be highly relevant in workers' compensation claims. Hence a reliable method for a valid retrospective estimation of cumulative ETS exposure is necessary.

Valid risk assessment of health effects caused by ETS is difficult due to problems with proper exposure assessment as well as it being necessary to restrict the group of exposed persons to the subgroup of those who have never smoked. So far smokers' exposure to ETS was thought to be negligibly low compared to the amount inhaled by active smoke. This opinion might change in future as a recent study from Piccardo et al. found that ETS exposure in active smokers seems be important at least in indoor environments [14]. However, at present the risk increase due to ETS in smokers and ex-smokers appears to be non-definable. Furthermore, as ETS occurs at work as well as at home or during leisure, the concentration and intensity of specific ETS indicators varies spatially and temporally. The long latency period between exposure and diseases like lung cancer, compounds the problem as epidemiological studies have to estimate exposure over a long time period.

Studies dealing with decomposition products of nicotine (like cotinine and nicotine in urine, saliva and blood) as indicators for acute ETS exposure are readily available [15-26]. Further studies investigated whether these indicators produce valid measurements of ETS exposure [20,27-46].

In contrast, valid retrospective exposure assessment of chronic ETS exposure is especially difficult as no surveillance data exists. Most existing studies used questionnaires for exposure assessment. However, these were only validated in terms of acute exposure [47,48]. The questionnaires mostly included items like number of smoking colleagues at work [49], duration of daily exposure and number of years at work [50].

Due to the subjectivity of this method it can not be used directly in compensation claims in Germany. Furthermore, the fact that exposure might change over years and smokers might be misclassified as non-smokers would probably complicate the acceptance of a legally defined occupational disease due to ETS exposure.

Therefore this literature-based methodological review aims for a reliable model for retrospective exposure assessment of ETS in the hospitality setting. This method should be used in the future for the determination of exposure thresholds in German workers' compensation claims.

## Methods

### Literature-based methodological review

A systematic literature-based methodological review was carried out using the Medline database with the following selection criteria:

Keywords:

• "tobacco smoke pollution" as a generic term for ETS (Medline Search Term-MeSH, including the terms "ETS", "second hand smoke" and "passive smoking")

• "restaurant" as generic term for hospitality setting (including the terms "disco", "bar", "tavern" and "pub")

• "occupational exposure" or "risk assessment" for exposure and risk assessment

The keywords were combined in the following way:

(("Tobacco Smoke Pollution"[Mesh] AND (((("Restaurants" [Mesh]) OR "Risk assessment" [Mesh]) OR "occupational exposure" [Mesh]))).

Using the function "limits", further selection criteria were chosen:

• Publication language: German and English

• Time period: 1990 to June 2007

• This time period was selected due to the fact that before 1990 most studies focused on ETS exposure at home.

This method generated 575 publications. First the abstracts of all 575 publications were screened; 94 abstracts indicated that they addressed the above mentioned aims of this review and were included in the further analysis. The remaining 481 publications were excluded from further analysis mostly because they contained the following:

• health effects of active and passive smoking in children, adolescents and adults in general

• health effects of maternal smoking and the embryonic development of their children

• methods, results, advantages and disadvantages of non-smoking programmes as well as attitudes of hospitality workers and guests towards these programmes.

• possible influences of the tobacco industry on medical research

• development of new methods for quantification of acute and subacute ETS exposure like DNA-adducts.

The remaining 94 articles were reviewed in full. Since all publications using a mathematical model for retrospective exposure assessment were based on a model developed in the 1980s, a further 15 publications were included [20,51-64] despite their earlier date of publication. Further publications were found in the bibliographies of the selected papers (n = 24).

87 publications of these133 publications were excluded for the following reasons:

• Exposure- and risk assessment did not deal specifically with the hospitality setting (N = 33)

• Comments and critical evaluation of existing publications and methods (N = 13); these were implemented in the discussion only

• Outcomes which are not relevant for the present question (e.g. acute symptoms of ETS exposure) (N = 8)

• Discussion of different methods of measurements regarding estimation of acute ETS exposure (N = 21)

• Publications dealing with advantages and disadvantages of different methods of measurements of acute ETS exposure (n = 12).

The remaining 46 publications were divided into the three categories mentioned (Table 1).

**Table 1:** Classification of the selected publications

Category	Number of publications [References]
1. Models for retrospective exposure assessment of ETS at the workplace as well as studies used for the development of these models	22 [51-64,70-76,111]

2. Publications dealing with acute exposure assessment especially in the hospitality industry	20 [79,81-98,112]

3. Retrospective exposure and risk assessment of diseases associated with ETS exposure in workers of the hospitality industry	4 [13,99-101]

The following information was extracted from each study if available:

• number and type of participants

• origin of study

• workplace (hospitality setting, others)

• investigated diseases

• air measurements: results for nicotine

• relative or absolute risk for the development of lung cancer due to ETS.

Only few authors restricted their analysis to persons who have never smoked, whereas the definition of those varied from the consumption of 0 and 100 cigarettes [65,66] up to 400 cigarettes [67,68] over a lifetime. All other studies used non-smokers or merged never- and ex-smokers into non-smokers. This classification is based on the hypothesis that the risk for lung cancer due to ETS exposure is no longer increased significantly after a lag period of 15 years [50].

## Results

### Models for calculation of indoor ETS exposure

#### Calculation of "cigarette equivalents"

Puntoni et al. [69] developed a mathematical model for the calculation of lung cancer risk at low cigarette consumption (< 5 cigarettes/day). By extrapolating data of nine cohort studies, the authors showed that there is a linear correlation between ETS exposure and lung cancer, even at low-dose low-level exposure. Based on these models Puntoni et al. calculated "cigarette equivalents" for ETS, converting ETS exposure to a corresponding number of actively smoked cigarettes. The cotinine plasma concentration of passive smokers was defined as 0.6-1.0% of the cotinine plasma concentration of active smokers [64]. The daily mean consumption of 20 cigarettes of active smokers would therefore be equivalent to 0.12-0.20 cigarettes/day of ETS for a passive smoker.

#### Models for retrospective exposure assessment

Mathematical models for retrospective exposure assessment are predominantly based on a model by Repace and Lowrey [58-61,70]. This model was developed for assessing the risk of lung cancer caused by ETS and thoroughly validated [71,72].

##### Estimate of nicotine exposure in the workplace

Repace and Lowrey assumed that ETS exposure is proportional to the smoker density (D_R_) and the inversely proportional to the air exchange rate(Φ_v_) measured as air changes per hour [58,59]. The smoker density is hereby based on the mean number of smokers in a room in relation to the room's volume and the mean number of cigarettes smoked per hour and per smoker. Repace and Lowrey used data from epidemiological studies in the USA, giving a smoking prevalence of 25% and the average number of smoked cigarettes of 2/h [72].

For example, a restaurant with an area of 100 m^2^, 70 available seats and a ceiling height of 4 m results in a room volume of 400 m^3^. With a smoker prevalence of 25% active smokers, the smoke density is as follows (Equation 1):

(1) $$Smoke\;density\;D_{R}=\text{7}0\times 0.\text{25}/\text{4}00=\text{4}.\text{5 smoker}/\text{1}00{\text{m}}^{3}$$

Nicotine concentration in indoor air was, although not representing a carcinogen, used as an indicator for ETS exposure [60] as it correlates with ETS exposure. However, the model was validated using other ETS indicators as well. Nicotine concentration (μg/m^3^) at the workplace was calculated as a function of smoke density D_R _(smoker/100 m^3^) and air exchange rate Φ_v _(air-changes/hour) (Equation 2).

(2) $$\begin{array}{l} Nicotine\;concentration_{ETS}\left(\mu g/m^{3}\right)=\\ {}[(\text{Nicotine emission per cigarette}\times \text{number of smoked cigarettes per hour}\times 0.\text{8 due to deposition on subjects})\times {\text{ smoke density D}}_{\text{R}}]/\text{Air exchange rate }\Phi_{\text{v}} \end{array}$$

Mean nicotine emission is 1400 μg nicotine/cigarette based on laboratory measurements.

The air exchange rate is based on the number of persons per room multiplied by the statutory ventilation rate (for a US restaurant 10l/second/person). This product is divided by the room volume in m^3^. In the example stated above this would result in 6.25 air exchanges/hour (Equation 3).

(3) $$\begin{array}{l} Air\;exchange\;rate\;\Phi_{v}\\ =\left(\text{7}0\text{ persons}\times \text{1}0\text{l}/\text{s}/\text{Person}\times {\text{1m}}^{3}/\text{1}000\text{l}\times \text{36}00\text{s}/\text{h}\right)/\text{4}00{\text{m}}^{3}=\text{6}.\text{25 exchanges}/\text{hour} \end{array}$$

Using equation 2 this example leads to

(4) $$\begin{array}{l} Nicotine\;ETS\left(\mu g/m^{3}\right)\\ =\left[\left(\text{14}00\mu \text{g}\times \text{2}\times 0.\text{8}\right)\times \text{4}.\text{5 smokers}\right]/\text{1}00{\text{m}}^{3}/\text{6}.\text{25}/\text{h}\;\;=\text{15}.\text{7}\mu \text{g}/{\text{m}}^{3}. \end{array}$$

The results of this formula were validated with measurements and showed good agreement [58,60]. To retrospectively calculate the exposure of an individual using these equations, certain parameters like company data (ventilation, number of guests), data from epidemiological studies (smoking prevalence in the population) and laboratory data (mean nicotine emission of a cigarette) are required (Table 2).

**Table 2:** Required parameters for Repace and Lowrey's model regarding retrospective exposure assessment of air nicotine concentration

Parameter (Unit)	Data source
Room volume V (in m^3^)	Fixed size for each hospitality environment

Number of guests/seats (in persons)	Mandatory legal standards define the maximum occupancy

Ventilation rate (in l/s pro Person)	In Germany: E DIN EN 15251/ASR (defined standard) for uninhabited buildings

Nicotine emission per cigarette (in mg nicotine per cigarette)	Means could be derived from measurements and studies of the accordant time period

Loss by nicotine deposit on surfaces (in percent)	Known physical parameter (80%) [58]

Number of smoked cigarettes per smoker (in/h)	Epidemiological data for observed time period

Smoker prevalence (in %)	Epidemiological data for observed time period

##### Further exposure models

Klepeis [73] investigated if indirect methods could lead to a valid estimation of ETS exposure in everyday life. Furthermore, Klepeis and colleagues [74,75] developed models regarding the dissemination of smoking sources. Based on these results, ETS produced by one cigarette will disperse within 12 to 80 minutes throughout an entire room. Similarly time exposure models were developed by Ott et al. [76] and in general these models for retrospective exposure assessment based on the physical law of conversation of mass were very similar. They were validated in experimental tests and epidemiological studies. An overview of the different models and their derivation is given by Ott [77].

##### Estimate of lung cancer risk

Repace and Lowrey also used their exposure model for the calculation of lung cancer risk caused by ETS exposure [2,59,70,71]. According to the publications of Doll and Peto [53], Puntoni [69] and other authors [51,52] they assumed a linear dose-response relationship. The baseline risk for lung cancer in non-ETS exposed persons who have never smoked was derived from epidemiological studies [56,57,70,72]. Based on these results Repace and Lowrey calculated the additional lung cancer risk at the workplace [72] and applied it to the hospitality setting [71]. Basic lung cancer risk in the USA was given as 7 cases/1000 persons amongst non-smoking unexposed women [71,78].

For risk calculation of occupationally exposed workers, duration of employment and daily working hours were taken into account. Repace and Lowrey assumed 40 years of employment with 260 working days per year and 6.7 working hours per day. Respiratory minute volume was defined as 1 m^3^/h for physically easy work. According to the above calculations, this results in one additional death from lung cancer per 7.5 μg/m^3 ^of nicotine in the air in 1000 employees [71,72] (Table 3).

**Table 3:** Calculation of additional deaths given an occupational exposure to ETS of 40 years based on the model by Repace und Lowrey in comparison to the baseline risk [71].

Country	Place of ETS exposure	**Estimated nicotine exposure (μg/m**^**3**^**)**	Number of lung cancer deaths per 1000 persons
**USA**	No exposure	0	7
	Only domestic exposure	4.3	10
	Office	11.2	11.5* (8.5)#
	Restaurant	15.7	12.0* (9.0) #
	Smoker club	20.3	12.7* (9.7) #

Data for USA. (* Occupational and domestic exposure, # only occupational exposure)

However, it should be borne in mind that the epidemiological data for calculating the additional deaths is based on data from the USA. For a reliable calculation in other countries, equivalent epidemiological data should be used.

### Acute ETS exposure in the hospitality environment

As stated above, acutely measured nicotine concentration is, especially after implementation of the smoking ban, not suitable for estimating chronic ETS exposure. However, these studies are used to illustrate the typical exposure level [79-98] (additional file 1). As expected the results of these studies vary largely, most likely due to different exposure situations in the individual countries. Bohannon and colleagues [82] showed that mean nicotine air concentration in six different countries could vary up to a factor of 10. In Germany, Bolte et al. found nicotine concentrations ranging from 71 to 450 μg/m^3 ^in discos [83]. After inputting this data into Repace and Lowrey's model, a lifetime risk of developing lung cancer of 16.5 to 67.0 per 1000 persons due to occupational ETS exposure is calculated. The large variation of these results outlines the importance of a valid retrospective exposure assessment.

### Risk assessment of diseases associated with ETS exposure in workers of the hospitality industry

In total, four studies regarding retrospective exposure and assessing the risk of workers in the hospitality industry were found. Hedley et al. [99], Mulcahy and Repace [100] as well as Siegel and Skeer [13] used the above mentioned model of Repace and colleagues [2,59,72] for studies in Hong Kong [99], Ireland [100] and the USA [13]. The Jamrozik's approach [101] was different, estimating risks attributable to ETS in the hospitality industry in Great Britain.

#### Hospitality workers in Hong Kong

Hedley et al. [99] used Repace and Lowrey's model [58,59,72] for calculating the additional risk of developing lung cancer and heart disease caused by ETS exposure at the workplace. Urinary cotinine concentrations were converted to ETS exposure as mentioned in Repace and Lowrey's model. According to their model [2,61], lung cancer and heart disease mortality for non-smoking hospitality workers in Hong Kong increased by 30 cases per 1000 workers due to ETS exposure over 40 years. The authors stated a ratio of 10:1 (cardiac events to lung cancer) resulting in 3 additional deaths per 1000 workers due to lung cancer.

#### Hospitality workers in bars in Ireland

Mulcahy and Repace estimated mean saliva cotinine concentrations of 7.4 ng/ml based on air carbon monoxide measurements in 14 Irish bars [100]. Using Repace and Lowrey's approach [2], they calculated an additional lung cancer mortality of 18 per 1,000 for the 28,000 fulltime working bar staff in Ireland given 40 years of working time.

This study was published only in the form of congress proceedings and only a few details were given with regard to the approach used. Therefore, results have to be interpreted carefully.

#### Hospitality workers in the USA

Siegel and Skeer estimated the number of additional lung cancer cases in the hospitality industry in the USA [13]. They included current literature regarding nicotine exposure measurements in bars, billiard and bowling centres, bingo halls and restaurants in the USA (Table 4). Based upon the nicotine concentrations, the number of the additional lung cancer cases was calculated using Repace and Lowrey's model [2,59,72]. This resulted in an additional 1.0 to 4.1 lung cancer deaths per 1000 workers over a 40 year working period. As expected, mortality was associated with higher exposure concentrations (weighted mean nicotine concentration 31.1 μg/m^3^) and longer working shifts (40 hours/week) in bar staff.

**Table 4:** Additional deaths for occupational and private ETS exposure over 40 years based on the formula by Repace and Lowrey as well as epidemiological studies [71]

Study	Exposure	Weighted mean nicotine concentration in μg/m3	Lifetime lung cancer risk per 1000 workers (95% CI)	
			
			Men	Women
[71,78,109]	No exposure	Non applicable	5.6 (4.9 - 6.3)	4.5 (4.0 - 4.9)

	Only exposure at home	4.3	8.4 (7.4 - 9.5)	6.7 (6.0 - 7.4)

[83]		Range:	#	#

	Restaurants	0.7-	5.6 (4.9 - 6.3)-	4.5 (4.0 - 4.9)-
		83.3	16.7 (16.0 - 17.4)	15.6 (15.1-16.0)

	Bar/Pub	9.1-	6.8 (6.1-7.5)-	5.7 (5.2 - 6.1)-
		180	29.6 (28.9 - 30.3)	28.5 (28.0-28.9)

	Disco	71.0-	15.1 (14.4 - 15.8)	14.0 (13.5 - 14.4)-
		450	65.6 (64.9-66.3)	64.5 (64.0 - 64.9)

# Exposure only at workplace

#### Hospitality worker in Great Britain

Jamrozik [101] used a different approach for estimating the attributable risk of dying from lung cancer, coronary artery disease and stroke in the hospitality industry in UK Based on a study from New Zealand [102], he estimated a mean relative risk of 1.24 for lung cancer caused by ETS exposure at home. A study from Great Britain [21] projected an increase in exposure by 3.04 times in non-smoking hospitality workers in comparison to people only exposed at home, resulting in a relative risk of 1.73 for lung cancer, 1.61 for coronary heart disease and 2.37 for strokes amongst workers in the hospitality industry. Based on this data, the author estimated that 54 annual deaths were caused by ETS in the UK hospitality sector, 15 of which from lung cancer.

## Discussion

The aim of this publication was to review the existing literature on ETS in order to determine a reliable model for retrospective exposure assessment of ETS in the hospitality industry.

Neither questionnaires nor markers of acute exposure could be used in this context due to potential lack of validity of self-reported exposure [103].

The review indicated that a model by Repace and Lowrey which had already been developed in the 1980s might best serve this purpose [2,13,58,61,70-72,99,100]. By applying different parameters (room volume, ventilation and mean number of smoked cigarettes per hour), ETS exposure in the hospitality industry can be estimated. Using this model, the additional risk for lung cancer due to ETS exposure at work can be calculated taking the exposure duration into account.

### Methods

The choice of keywords in this literature review was based on the given problem. By using a combination of several MeSH-terms, it was ensured that the search results were as complete as possible. The study population was not restricted to hospitality workers as exposure assessment models from other sectors might have been used [13,71,99-101]. Due to the fact that before 1990 ETS exposure was mostly investigated at home, publications prior to this date were initially excluded. However, it was noticed that the available studies dealing with ETS exposure in the hospitality industry referred to a model which was already developed in the 1980s. Hence an additional 15 publications which had been shown to be relevant to the topic were included [20,51-64].

### Results

#### Cigarette equivalents

Puntoni et al. [69] developed a model for calculating lung cancer risk due to ETS exposure at low cigarette consumption levels (< 5 cigarettes/day). Several studies showed that a linear correlation to estimate the dose-response relationship between ETS and lung cancer is applicable [51-53,69]. The linear correlation was questioned in some publications [104,105]. However, it must be considered that financial support from the tobacco industry could have influenced the objectivity of this author [106].

#### Further models for retrospective assessment of ETS exposure

All further models for retrospective assessment of ETS exposure at work and in the environment were based on the model published by Repace and Lowrey. Other models, which assumed life-long smoking habits [76,77] found results consistent with Repace and Lowrey, who had used epidemiological data regarding the retrospective assessment of smoking habits in the hospitality industry. Several epidemiological studies and Monte Carlo simulations validated this model [2]. However, it has to be taken into account that nearly every non-smoker is exposed to ETS in daily life. Due to the fact that the average occupational exposure is much higher in the hospitality industry than at home, the additional risk seems to be clearly definable. Nevertheless, it has to be taken into account that mathematical models are only approximations of real exposure, especially if the latency period between exposure and disease is long.

#### Conversion of exposure amount in additional lung cancer risk due to ETS exposure in the hospitality industry

Repace and Lowrey [2,59,72] defined a lifelong working time of 40 years as the basis for calculating the additional lung cancer risk due to ETS exposure at work. Mulcahy and Repace [100] reported that only 45% of bar workers are employed in this sector for more than 10 years, whereas in other areas, such as discos, the working time could even be shorter. This has to be taken into account when calculating the additional number of lung cancer deaths. In contrast to the individual number of working years, assessment of the individual mean weekly working time could be difficult due to the high frequency of part time jobs in the hospitality industry.

This literature-based methodological review shows that in general the model of Repace and Lowrey is suitable for retrospective exposure assessment of ETS exposure in the hospitality setting [13,71,100,107]. The resulting exposure calculated using this model reflects the real exposure validly, as demonstrated in a meta-analysis by Stayner et al [10]:

Using Repace and Lowrey, an additional lung cancer death per 7.5 μg/m^3 ^nicotine at the workplace arises given employment duration of 40 years in the USA. Using Repace and Lowrey's formula, the predicted nicotine exposure at an office workplace is 11.2 μg/m^3^. This leads to 8.5 lung cancer deaths per 1000 workers without private exposure in comparison to 7/1000 for workers without occupational and private exposure and an RR of: RR = 8.5/7 = 1.21.

In comparison with the total RR (relative risk) of 1.24 (95% CI 1.18-1.29) for occupationally exposed people reported by Stayner et al. [10], the calculation by Repace and Lowrey lies within the 95% confidence interval of the results by Stayner et al.

However, type of occupation is important. For the most highly exposed people a RR of 2.01 (95% CI 1.33-2.60) was shown in this meta-analysis. Using data from a review by Siegel [96] with nicotine concentrations ranging from 7.4 to 65.5 μg/m^3 ^in bars, a relative risk of 1.14 to 2.25 can be calculated. These results would suggest that Repace and Lowrey's calculation may even underestimate the real relative risk.

However, the transfer of these methods from a population to individuals is difficult [108]. One of the major problems is the small number of non-smokers with lung cancer [109]. This results in studies based on small samples and thus large confidence intervals. Furthermore, the wide spread of measurements demonstrates the differences in the extent of workplace exposure and emphasizes the importance of individual exposure assessments.

#### Assessment of acute ETS exposure by air nicotine concentration in the hospitality industry

The validity of Repace and Lowrey's model was illustrated in a summary of studies using air nicotine concentrations as a leading parameter for ETS exposure in the hospitality industry. However, other markers like total dust exposure or cotinine concentration in body fluids led to similar results [2,71]. The appropriate correlation of atmospheric and biological markers in studies of ETS exposure has been discussed in detail by Repace et al. [110].

A study by Bolte et al. [83] showed that prior to the implementation of the smoking ban the mean nicotine air concentration in restaurants, cafes, pubs and bars in Germany was comparable to other European studies [83,86,94,95,97]. In contrast, the nicotine air concentrations in discos and nightclubs were higher in Germany than in other European studies [83,86,94,95,97].

Hence exposure differences in different countries as well as in different hospitality environments have to be taken into account. Based on the nicotine concentrations detected in the hospitality industry in Germany before the smoking ban [83], the lung cancer risk of a non-smoking restaurant worker after 40 working years could be greater by a factor of 3. This factor could increase to 5.8 in bar workers and to 13.0 in disco employees, who represent the most highly exposed group (Table 4). Even though the underlying base risk was obtained from the USA, these numbers indicate the legal relevance of this problem.

This data is concordant with the relative risks reported in epidemiological studies (see above).

#### Retrospective exposure assessment and calculation of lung cancer risk for hospitality workers

The results presented here are based on lung cancer as an outcome, because for common diseases such as cardiac events the additional risk due to ETS cannot be clearly defined for workers' claims compensation in Germany.

Overall, the studies show a clear additional risk of lung cancer mortality in hospitality workers. Estimates based on Repace and Lowrey's model resulted in an additional risk for lung cancer mortality of 1 per 1000 workers in bowling alleys in the USA and 18 per 1000 workers in bars in Ireland. Here it has to be taken into account that different respiratory minute volumes were used. Hedley et al. used a respiratory minute volume of 1 m^3^/h in line with light physical work [99]. In the hospitality industry moderately heavy physical work can be assumed. Thereby the publication underestimates the real risk at 3 per 1000 workers.

The considerably higher risk shown in the publication of Mulcahy and Repace is due to the underlying higher exposure amount. Unfortunately, further details were not mentioned in this paper [100].

Jamrozik [101] calculated a relative risk of 1.73 for lung cancer due to ETS of workers in pubs, night clubs and bars. However, neither the derivation of the relative risk nor the confidence intervals were explained in detail.

## Conclusion

Prior to the introduction of a partial or full smoking ban in many countries, ETS exposure was a cause of health risks in hospitality workers who never smoked. Based on the available data, employees working in bars, pubs, discos and night clubs in particular are exposed to high levels of ETS.

The main problem in calculating the risk of lung cancer due to ETS exposure results from the long latency period between exposure and disease. Retrospective exposure assessment for workers' compensation claims can be based on mathematical models where direct exposure measurement is not feasible.

Repace and Lowrey's model uses retrospectively determined parameters like room volume, number of seats, ventilation rate as well as nicotine concentration per cigarette to calculate nicotine concentration as leading parameter for ETS exposure. Data regarding smoker prevalence and number of smoked cigarettes per smoker and hour can be obtained from epidemiological studies. If the working time is known, the additional lung cancer risk for exposed workers can be calculated and compared with epidemiological data of basic lung cancer risk of non-smokers.

## Abbreviations

DNA: Desoxyribonucleic Acid; ETS: Environmental Tobacco Smoke; MeSH: Medline Search Term; RR: Relative Risk; USA: United States of America; UK: United Kingdom

## Competing interests

The authors declare that they have no competing interests.

## Authors' contributions

DN and KR conceived the study and helped to draft the manuscript. SK and UB organised the literature-based methodological review. SK drafted the manuscript. All authors read and approved the final manuscript.

## Supplementary Material

Additional file 1 **International studies for assessing acute air nicotine exposure in the hospitality setting**: The provided table shows international studies dealing with the assessment of acute air nicotine exposure in the hospitality setting. As expected the results of these studies vary largely, most likely due to different exposure situations in the individual countries. Click here for file
