# Supplementary material for: Quantification of ETS exposure in hospitality workers who have never smoked
Source: Environ Health. 2010 Aug 12;9:49. doi: 10.1186/1476-069X-9-49 (PMC2933666; doi:10.1186/1476-069X-9-49)
Supplement: Additional file 1 — International studies for assessing acute air nicotine exposure in the hospitality setting: The provided table shows international studies dealing with the assessment of acute air nicotine exposure in the hospitality setting. As expected the results of these studies vary largely, most likely due to different exposure situations in the individual countries. [file 1476-069X-9-49-S1.DOC]

# Quantification of ETS exposure in hospitality workers who have never smoked

**Additional file 1: International studies for assessing acute air nicotine exposure in the hospitality set**ting

| **Primary author** | **City, Country** | **Workplace (n measurements)** | **Mean duration of measurement (range) in hours** | **Location of  measurement  (n measurements)** | **Nicotine in µg/m^3^ mean (range)** |
| --- | --- | --- | --- | --- | --- |
| 1. Akbahr-Khanzahdeh [78] | Toledo, USA | restaurants :   NS area (n=13)  S area (n=16) | 8 | stationary | 7.2 (0.1-54.4) 24.9 (0.5-121.7) |
| 1. Akbahr-Khanzahdeh [79] | Toledo, Bowling Green, USA | NS restaurants (n=4) S restaurant: NS area (n=5) S area (n=5) | 6,4 (5,4-7,85) | stationary | 0.08 (SD 0.02)  1.75 (1.63) 14.2 (15.3) |
| 1. Bergman [80] | Oklahoma, USA | nightclub (n=9 musicians) | 18,5-29,5 | Passive sampler worn by musicians | 37.1  (28.0-50.0) |
| 1. Bohanon [81][[1]](#footnote-2) | France (n=15) Japan (n=16) Korea (n=47) Switzerland (n=32) UK (n=20) USA (n=18) | restaurants | 3-4 | Stationary  measurements at  2 locations, in case of NS areas:  1x S[[2]](#footnote-3) area, 1x NS area | 30.3 (0.0 – 71.6) 11.7 (3.4-22.4) 5.7 (1.6-18.8) 7.8 (0.1-39.6) 9.78 (0.8-27.6) 3.03 (0.04-9.43) |
| 1. Bolte [82] | Oberschleißheim,  Germany | restaurants (n=11) pubs (n=7) discos (n=10) | 4 | stationary | 21.3 (0.7-83.3) 53.7 (9.1-180.0) 226.6 (71.0-450.0) |
| 1. Cenko [83] | Adelaide, South Australia | bars, restaurants, game hall  NS area (n=10)  S area(n=7) | 2-4 | stationary | 7 (<0.1 – 21.5) 15 (3 – 38.8) |
| 1. Collet [84] | Vancouver, Canada | nightclubs (n=13) taverns (n=8) pub (n=10) | 2x1 h | stationary | 58. 0 (21.8-119.5) 46.6 (29.5-68.6) 38.6 (6.3-63.9) |
| 1. Ellingsen [85] | Norway | Before smoking ban (n=58)  restaurants (n=14)  bars (n=44) After smoking ban | One evening shift | stationary at 4-6 places per company | 28.3 (0.4-88.0) 7.7 (0.4-18.2) 34.9 (8.1-88.0) 0.6 (<0.01-3.7) |
| 1. Gee [86] | Manchester,  GB | bars and pubs | 4 | stationary in bar area (n=81) stationary in S area (n=134) stationary in NS area (n=23) | 77.1 ((IQ Range 25.4-93.4)  88.2 (IQ Range 23.7-132.3)  27.1 (IQ Range 10.6-42.7) |
| 1. Hyvärinen [87] | Helsinki, Finland | nightclubs/discos (n=4) pubs (n=3) restaurants (n=3) | 4 | Passive sampler worn by service staff | 42.2 (SD 6.3) 8.2 (SD 2.0) 7.0 (SD 2.3) |
| 1. Jenkins [88] | Knoxville  USA | taverns restaurants | 5,8 (bartender);  4,9 (service staff) | Active sampler worn by bartenders (n=80)  service staff(n=85) | Median 22.4 µg-h/m^3^ Median 5.6 µg-h/m^3^ |
| 1. Johnsson [89] | Helsinki, Finland | nightclubs, discos. (n=21) pubs (n=48) restaurants (n=16)[[3]](#footnote-4) | 7 (4-14) 7 (3,5-10) 7 (5-11) | Active sampler worn by  service staff | 10.2 (1.1-36.0) 3.7 (0.05-39.0) 1.4 (0.03-6.2) |

| 1. Johnsson [90] | Finland | pubs, night clubs,  restaurants, bars | 4 | Stationary (n=20) Before implementation of the NS protection law (n=20)  After implementation of the NS protection law [[4]](#footnote-5) (n=18) | GM 7.3 (GSD 5.8)    GM 7.1 (GSD 7.9) |
| --- | --- | --- | --- | --- | --- |
| 1. Kuusimäki [91] | Helsinki, Finnland | restaurants, bars und cafés in NS area (n=50) | ≥ 8 | stationary , 2 passive sampler per station | 0.7 – 85 |
| 1. Maskarinec [92] | Knoxville,  USA | restaurants (n=32) bars (n=53) | ≥ 3 | stationary | 6.01 (0-49.3) 14.4 (0-61.3) |
| 1. Mulcahy [93] | Galway, Ireland | bar (n=20) | 7 - 10 | stationary in bar area Before implementation of the NS protection law: After implementation of the NS protection law: | 35.8 (SD 25.7)  10.23 (SD 9.66) |
| 1. Nebot [94] | Vienna, Austria   Paris, France   Athens, Greece  Florence, Italien   Oporto, Portugal Barcelona, Spain  Örebro, Sweden | bars, discos (n=22) restaurants S area (n=10)   NS area (n=7) bars, discos (n=3) restaurants S area (n=12)  NS area (n=6) restaurants S area (n=7) bars, discos (n=8) restaurants S area (n=17)  NS area (n=3) restaurants S area (n=6) bars, discos (n=3) restaurants S area (n=8) bars, discos (n=2) restaurants S area (n=14)  NS aea (n=8) | 4 h - 14 day | stationary | Median:  122  17 18 59 9.3 1.6 4.7 19 1.6 2.2 0.01 91 7.8 < 5 7.1 0.1 |
| 1. Siegel [95] | Review | bar restaurants office home | NA[[5]](#footnote-6) | NA | 19.7[[6]](#footnote-7) (7.4-65.5) 6.5 (3.4-34) 4.1 (0.8-22.1) 4.3 (1.6-21)) |
| 1. Skogstad [96][[7]](#footnote-8) | Oslo, Norway | bars, restaurants (n=13) | One night shift | before smoking ban after smoking ban | 28.3 (3-65) 0.6 (<0.01-1) |
| 1. Trout [97] | Cincinnati, USA | casino | 7 | stationary | 6-12 |

1. Different laboratories in each country [↑](#footnote-ref-2)
2. S = Smoker, NS = Nonsmoker [↑](#footnote-ref-3)
3. Number of measurement days [↑](#footnote-ref-4)
4. NS law only implemented to a limited extent; NS areas as well as pure smoking restaurants possible [↑](#footnote-ref-5)
5. NA Not applicable [↑](#footnote-ref-6)
6. Weighted mean [↑](#footnote-ref-7)
7. Part of the data set of number 8 [↑](#footnote-ref-8)
